# Supplementary material for: Nonsequential Splicing Events Alter Antisense-Mediated Exon Skipping Outcome in COL7A1
Source: Int J Mol Sci. 2020 Oct 18;21(20):7705. doi: 10.3390/ijms21207705 (PMC7590164; doi:10.3390/ijms21207705)
Supplement: Supplementary file 1 [file ijms-21-07705-s001.pdf]

Supplementary Material

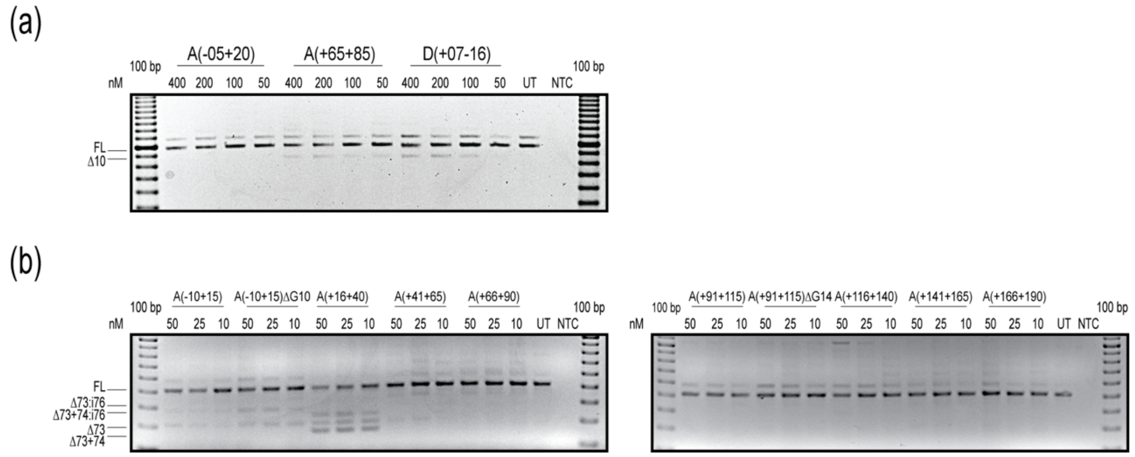

Figure 1. Evaluation of antisense oligomers (AOs) targeting *COL7A1* exons 10 and 73 after transfection into healthy human fibroblasts. (**a**) Reverse transcription-PCR analysis of *COL7A1* transcript after transfection with 2′-OMe AOs targeting *COL7A1* exon 10, transfected at concentrations indicated above the gel image. (**b**) Reverse transcription-PCR analysis of *COL7A1* transcript after transfection with 2′-OMe AOs targeting *COL7A1* exon 73, with transfection concentrations indicated above the gel image. NTC, no template control; UT, untreated; bp, base pairs; FL, full-length amplicon; nM, nanomolar.

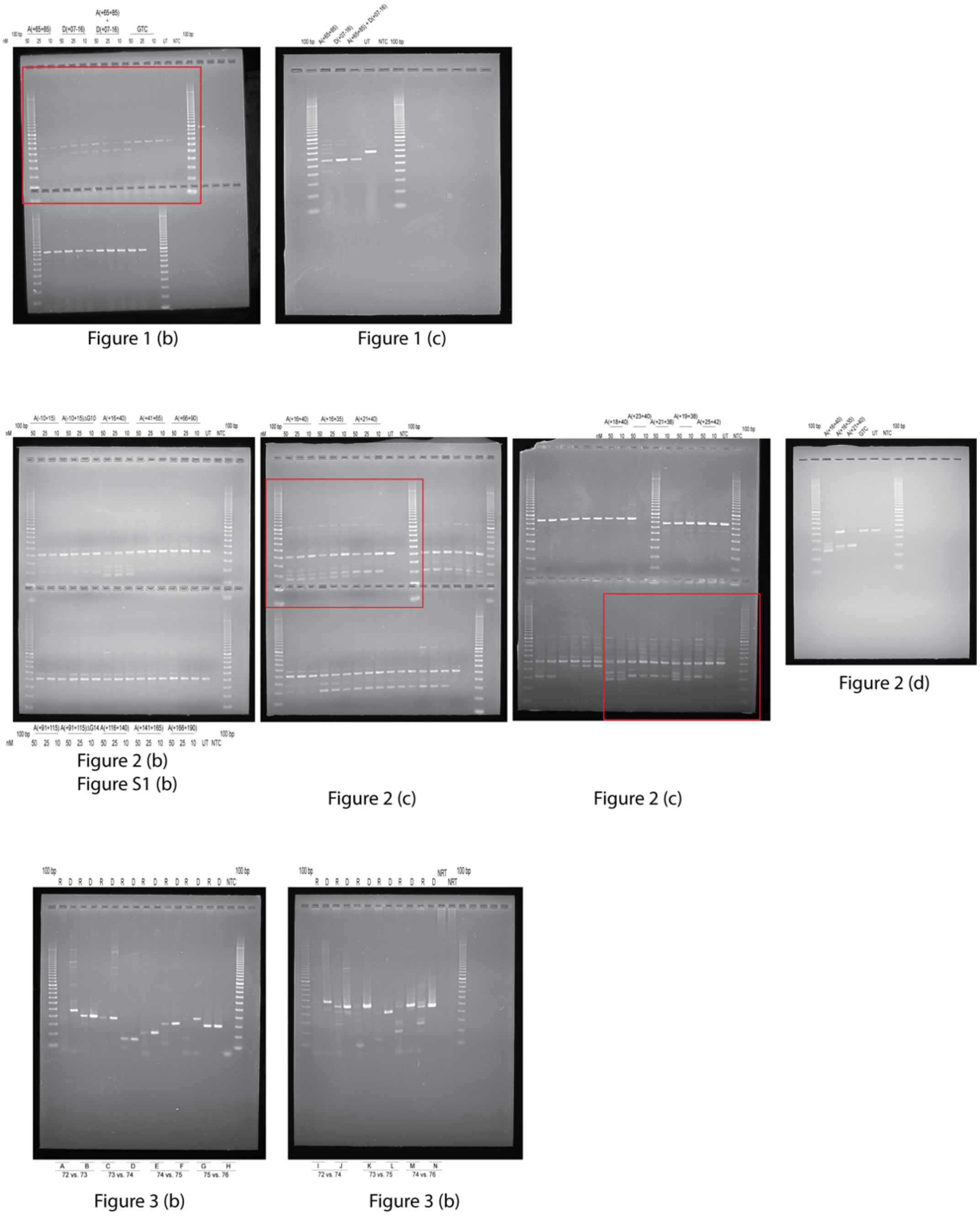

Figure 2. Full gels for figures listed.
